# Nitrogen-Doped Carbon Quantum Dots as Fluorescent Probes for Sensitive and Selective Detection of Nitrite

**DOI:** 10.3390/molecules22122061

**Published:** 2017-11-24

**Authors:** Zhibiao Feng, Zeliang Li, Xingwei Zhang, Yanping Shi, Nan Zhou

**Affiliations:** Department of Chemistry, Northeast Agricultural University, Harbin 150025, China; fengzhibiao@neau.edu.cn (Z.F.); lizeliang123@aliyun.com (Z.L.); zhangxingwei123@aliyun.com (X.Z.); aliali12345@aliyun.com (Y.S.)

**Keywords:** nitrogen-doped carbon quantum dots, fluorescence probe, nitrite measurement, quenching mechanism

## Abstract

Nitrites are the upstream precursors of the carcinogenic nitrosamines, which are widely found in the natural environment and many food products. It is important to develop a simple and sensitive sensor for detecting nitrites. In this work, a fluorescence probe based on nitrogen-doped carbon quantum dots (N-CQDs) was developed for the sensitive and selective determination of nitrites. At pH 2, the fluorescence of N-CQDs can be selectively quenched by nitrite due to the fact N-nitroso compounds can be formed in the reaction of amide groups with nitrous acid, which results in fluorescence static quenching. Under optimal conditions, fluorescence intensity quenching upon addition of nitrite gives a satisfactory linear relationship covering the linear range of 0.2–20 μM, and the limit of detection (LOD) is 40 nM. Moreover, this method has been successfully applied to the determination of nitrites in tap water, which indicates its great potential for monitoring of nitrites in environmental samples.

## 1. Introduction

Nitrosamines are formed from nitrites and are known carcinogens that have toxicological effects on humans [1]. Nitrosamines are found in the natural environment, and also in many food products [2]. Hence, it of is of great significance and a necessity to determine the presence of nitrites and to provide sensitive and selective assays for their early detection. Numerous methods for detecting and determining nitrite have been reported using ultraviolet-visible (UV-vis) spectrophotometric [3], chemiluminescent (CL) [4,5,6], electrochemical [7,8] and spectrofluorimetric methods [9,10]. Ultraviolet spectrophotometry is the most commonly used method for the detection of nitrite. Diazonium compounds can be formed during the reaction of a primary aromatic amine with nitrous acid. The absorbance of the product after this reaction is proportional to nitrite concentration. However, this method is greatly limited because of its poor sensitivity and interferences from other participating ions [11]. Although, nitrite is electroactive at platinum, gold, copper, diamond, and transition metal oxide electrodes [12,13,14], electrochemical methods are not preferred for trace analysis due to their poor selectivity. Nitrites can be detected in gaseous or aqueous phase systems using CL methods. In the former case, nitrites are reduced to nitrogen oxide by a reductant and NO_2_* is formed by the subsequent reaction of nitrogen oxide with ozone, which is electronically excited and falls to a ground state with light emission [15]. For aqueous phase detection, nitrites react with H_2_O_2_ forming the intermediate peroxynitrous acid (ONOOH) which is further treated with alkali to form peroxynitrite. Since the decomposition of peroxynitrite is associated with CL emission, the flow-injection technique was developed for nitrite determination [16]. The intensity of CL emission is related to environmental factors and any change in these factors can adversely impact the stability and reproducibility of CL results [17].

The underlying principle on which spectrofluorimetric methods are based, utilizes the variations recorded in fluorescence intensity during the reaction between fluorescent probes and nitrite. This technique has been more commonly used to detect nitrite concentration because it provides high sensitivity, good selectivity, excellent limits of detection and comprehensive suitability. Several such probes have been developed by utilizing the chemical specificity of nitrite ion towards diazotization or nitrosation [17]. One of the probes, resorcinol, can react with nitrite to give nitroso derivatives, which cause a fluorescence intensity variation [10]. In these studies, the recorded changes in fluorescence intensity have been used for quantitative analysis of nitrite. Axelrod et al., have demonstrated an increase in fluorescence intensity during the reaction of 5-aminofluorescein with nitrite [18], however, it was found that the stability of such probes was pretty weak. This highlights the need for developing a stable, selective and robust probe.

Carbon quantum dots (CQDs) are small nanoparticles (less than 10 nm diameter) [19]. They were found to exhibit photoluminescent properties when first discovered accidentally by Xu et al. in 2004 [20]. CQDs present high chemical stability, bio-compatibility, and excellent optical properties, as well as ease of surface modification [21,22], and have already been widely applied in diverse fields, including cell imaging [23,24,25], biochemical sensing [26,27], and analysis [28]. In solution, the fluorescence intensity of CQDs can be quenched by an electron donor or an electron acceptor molecule, indicating that CQDs themselves are good electron donors or acceptors. By using this property, CQDs can assist in identifying certain specific ions in solution [29,30]. At present, very few published studies are available in the literature on the use of CQDs to detect nitrite. CQDs have chemiluminescent properties in the presence of ONOOH, formed by the reaction between H_2_O_2_ and NaNO_2_. Lin et al., developed an injection method for its detection, however, this requires special pumps and added hydrogen peroxide reagents [16]. Nitrogen-doped carbon quantum dots (N-CQDs) were prepared via carbonization of citric acid in the presence of triethylenetetramine as a nitrogen source, and were introduced as a novel fluorescence probe to determine NO_3_^−^ and NO_2_^−^ via their quenching behavior [31]. However, due to poor specificity, the method could not directly distinguish between NO_2_^−^ and NO_3_^−^ In the present work, N-CQDs have been applied for building a direct, fast and simpler nitrite detection method.

In this research, we present a fluorescent assay for nitrite detection by using N-CQDs as fluorescence probes. N-CQDs were prepared by hydrothermal treatment of citric acid as the carbon source and EDA as the nitrogen source. At a pH of 2, the fluorescence of the N-CQDs can be selectively quenched by nitrite. A possible mechanism has been put forward whereby N-nitroso compounds can be formed in the reaction of amide group with nitrous acid, which result in fluorescence static quenching. Experimental results demonstrate that this proposed assay has robustness for the quantitative analysis of nitrite with high sensitivity, low cost and good selectivity. Furthermore, this method can also be applied for measuring nitrite in tap water samples.

## 2. Results and Discussion

### 2.1. Optimization of N-CQD Synthesis Conditions

**Figure 1:**
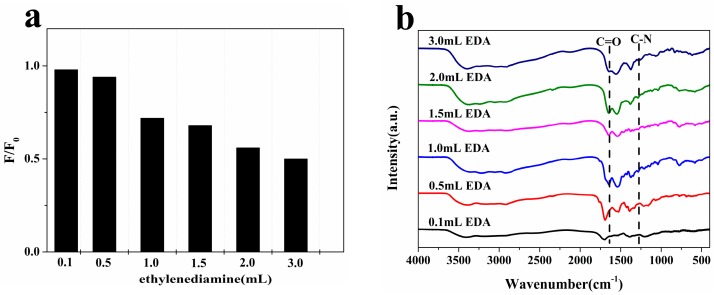
Effect of nitrogen source concentration on fluorescence properties of N-CQDs: (**a**) The degree of response to nitrite (10^−5^ M); (**b**) FTIR spectrum.

In order to optimize the N-CQD synthesis conditions, N-CQDs were synthesized with equal amounts of citric acid (3 g) as carbon source and different concentrations of EDA (0.1, 0.5, 1.0, 1.5, 2.0, and 3.0 mL) as the nitrogen source. Figure 1a shows the comparison of sensitivities for nitrite in samples synthesized with different concentrations of the nitrogen source. F_0_ and F represent the fluorescence intensity of N-CQDs at 480 nm in the absence and presence of NO_2_^−^. The initial samples with 0.1 mL and 0.5 mL on added nitrogen source had little response to nitrite, and as the amount of the nitrogen source was increased, the sensitivity of N-CQDs to nitrite gradually increased. In addition, the investigation on the surface structure of these N-CQDs through FTIR measurement, is shown in Figure 1b, which indicates that the N-CQDs synthesized with 0.1 mL and 0.5 mL of EDA have no amide groups, while the other samples have amide groups. The amide groups on N-CQDs contribute to the luminescent properties [32]. Combined with the results of ion sensitivity analysis (Figure 1a), the amide groups can be described as the major factor in the detection of NO_2_^−^. Therefore, the N-CQDs were synthesized with EDA (3 mL) as the nitrogen source and citric acid (3 g) as carbon source.

### 2.2. Characterization of the N-CQDs

**Figure 2:**
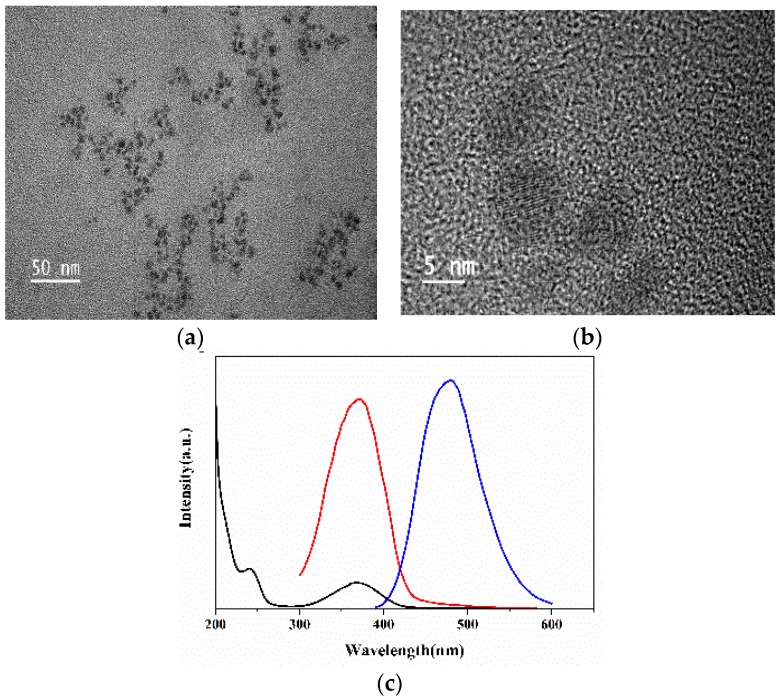
(**a**) TEM images of the N-CQDs; (**b**) Lattice structure of the N-CQDs obtained from HRTEM image; (**c**) UV-vis. absorption (**black line**); photoluminescence excitation spectra (**red line**) and emission spectra (**blue line**) of N-CQDs in aqueous solutions.

As shown in TEM images, N-CQDs are uniform in size (4–8 nm) and well dispersed in aqueous solution (Figure 2a). The HRTEM results revealed well crystallized N-CQDs with a lattice spacing of 0.241 nm, similar to a typical graphite structure (Figure 2b). According to the UV-vis spectrum, the absorption maxima was in the range of 300–400 nm.

The fluorescence spectra of the N-CQDs were further studied and a characteristic absorption band at 370 nm was found in the excitation spectrum that is consistent with the results obtained in the UV-vis study (Figure 2c).

**Figure 3:**
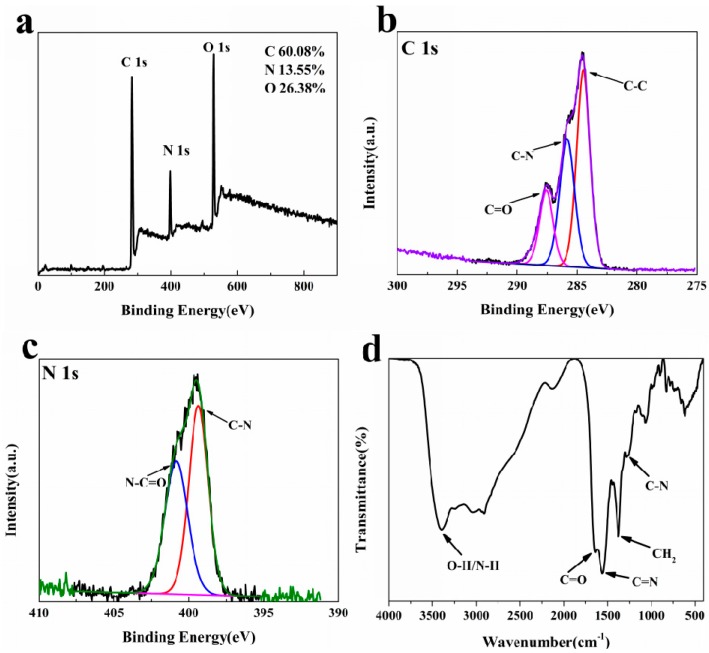
(**a**) XPS spectrum; (**b**) C1s spectrum; (**c**) N1s spectrum and (**d**) FTIR spectrum of the N-CQDs.

The XPS spectrum displays strong signals of C1s at 283 eV, N1s at 398 eV and O1s at 530 eV, with atomic percentages of 60.08%, 13.55% and 26.38% (Figure 3a). Deconvolution of the C1s spectrum reveals three peaks at 284.5, 285.8 and 287.6 eV, which are C-C, N-C and C=O groups, respectively (Figure 3b). The N1s spectrum can be resolved into two components at 399.4 and 400.8 eV respectively for N-C and O=C-N (Figure 3c) [33]. The surface structure of N-CQDs was investigated through Fourier transform infrared spectroscopy (FTIR) measurement. FTIR spectra were recorded to identify the functional groups on the N-CQDs. As can be seen in Figure 3d, the strong peaks at 1643 cm^−1^, 1554 cm^−1^, 1369 cm^−1^ and 1262 cm^−1^ were attributed to C=O, C=N, CH_2_ and N-C, respectively [34,35]. The broad band centered at 3386 cm^−1^ suggests the existence of O-H and N-H, for stability of N-CQDs in aqueous system indicating the presence of functionalized groups [36]. The results from XPS and FTIR have been validated to confirm N-CQDs are nitrogen-doped containing carbon-rich nanodots with active functional groups, such as hydroxyl, amide, amino and carboxyl/carbonyl moieties.

### 2.3. Feasibility of N-CQDs Based Sensor for NO_2_^−^

#### 2.3.1. Effects of pH on NO_2_^−^ Detection and N-CQDs

**Figure 4:**
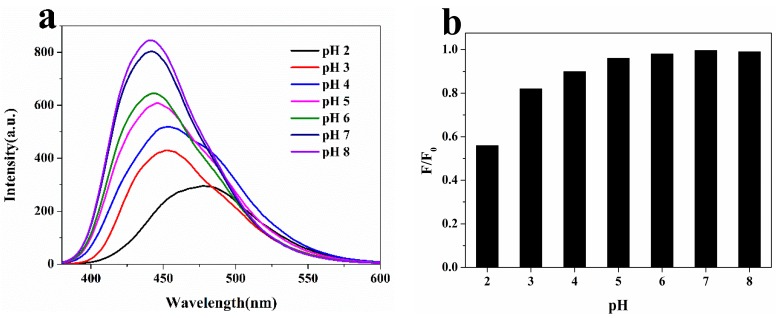
(**a**) The effect of pH (2.0, 3.0, 4.0, HCl-KCl buffer solution and 5.0, 6.0, 7.0, 8.0, PBS) on the fluorescence intensity of N-CQDs solution; (**b**) The effect of pH (2.0, 3.0, 4.0, HCl-KCl buffer solution and 5.0, 6.0, 7.0, 8.0, PBS) on the quenching of the fluorescence intensity of N-CQDs solution in the presence of nitrite (10^−5^ M) with a time duration of 15.0 min measured at room temperature.

As shown in Figure 4a, the fluorescence spectra were red shifted with the decrease of the pH value, while the fluorescence intensity decreased gradually. The effect of the pH values can be understood in terms of the change in surface charge owing to protonation–deprotonation [37]. Figure 4b shows that for the same concentration of N-CQDs and nitrite conditions, the degree or the level of quenching decreases with an increase in pH value. The nitrogen-containing groups and nitrous acid are usually reacted under strong acid conditions to produce N-nitroso compounds [38], which results in fluorescence static quenching. This indicates that fluorescence quenching is caused by chemical reactions. Hence, a pH of 2.0 is selected as the optimum value.

#### 2.3.2. Selectivity of N-CQDs Detection of NO_2_^−^

**Figure 5:**
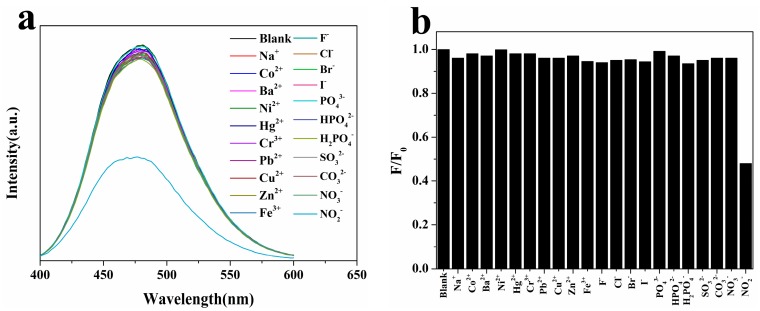
(**a**) Fluorescence spectra of the N-CQDs aqueous solution in the presence of different ions; (**b**) Fluorescence response of the N-CQDs aqueous solution in the presence of different ions at 480 nm.

Fluorescence screening experiments were performed for determining NO_2_^−^ and identifying potential interfering ions (Na^+^, Co^2+^, Ba^2+^, Ni^2+^, Hg^2+^, Cr^3+^, Pb^2+^, Cu^2+^, Zn^2+^, Fe^3+^, F^−^, Cl^−^, Br^−^, I^−^, PO_4_^3−^, HPO_4_^2−^, H_2_PO_4_^−^, SO_3_^2−^, CO_3_^2−^ and NO_3_^−^) within the N-CQDs aqueous solution (Figure 5a). Twenty kinds of biologically and environmentally relevant ions were used at a concentration of 10 μM (similar to the case of NO_2_^−^ excited at 370 nm and equilibrated time 15 min) to evaluate the changes in fluorescence intensity before and after the addition.

In Figure 5b, only the NO_2_^−^ ions significantly reduced the fluorescence intensity at 480 nm of the N-CQDs (quenching 52% of base case at 370 nm); the signal was slightly changed for the remaining ions. The mild quenching is attributed to interaction between metal and carboxyl group [39]. This result indicates that N-CQDs have a high selectivity for NO_2_^−^ detection.

#### 2.3.3. Sensitivity of N-CQD Detection of NO_2_^−^

**Figure 6:**
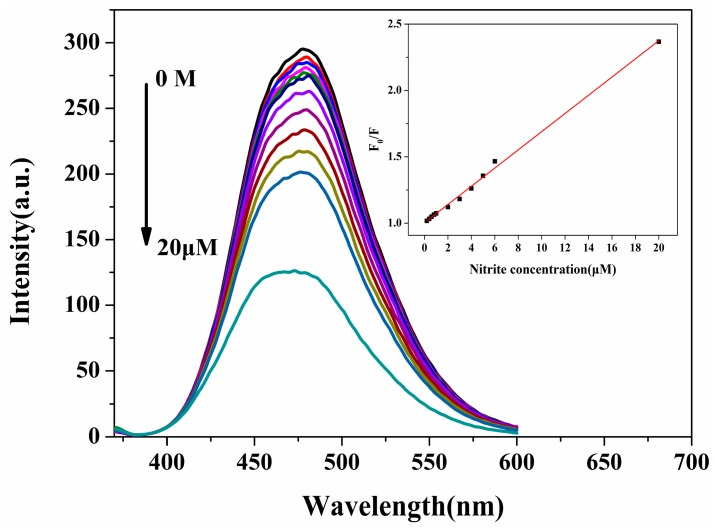
Fluorescence spectra of N-CQDs in the presence of different NO_2_^−^ concentrations (from **top** to **bottom**: 0, 0.2, 0.4, 0.6, 0.8, 1, 2, 3, 4, 5, 6 and 20 μM) in deionized water. The inset shows the dependence of F_0_/F on the concentrations of NO_2_^−^ within the range of 0–20 μM.

For susceptibility studies, fluorescence studies were performed to assess the response of N-CQDs to different NO_2_^−^ concentrations. As revealed in Figure 6, for N-CQDs, quenching is a function of intensity at 480 nm when reduced from 0.2 to 20 μM at 370 nm excitation. The Stern-Volmer equation for the quenching mechanism is given by Equation (1):

F_0_/F = 1 + K_sv_[Q] (1)

where K_sv_ is the Stern-Volmer quenching constant, [Q] is the concentration of quencher NO_2_^−^, and F_0_, F are the fluorescence intensity of N-CQDs at 480 nm in the absence and presence of NO_2_^−^, respectively. This equation fits the linear calibration plot over the entire NO_2_^−^ concentration range of 0.2–20 μM (inset of Figure 5). The slope of the calibration curve represents the Stern-Volmer constant, 0.068 (μM)^−1^, and the correlation coefficient (R^2^) is 0.9969. The LOD for NO_2_^−^ was calculated to be 40 nM using Equation (2):

LOD = 3σ/s
(2)

where σ is the standard deviation (σ = 0.09%) or repeatability of the response recorded for the blank N-CQDs sample (N = 10) and s is the slope. The LOD of 40 nM is remarkable, indicating N-CQDs are superior in detecting NO_2_^−^. Table 1 shows the evaluation of LOD in with different CQDs probes for NO_2_^−^. The method adopted in this work gives linear results and detection limits similar to the other methods used, as reported in literature, although those methods are more complex and require special reagents and instruments [16,40]. Doroodmand has determined the use of N-CQDs to detect nitrite, but was unable to directly distinguish between nitrite and nitrate [31]. In this paper, the method directly uses N-CQDs to detect nitrite, indicating the simplicity, rapid and selective performance of the method.

#### 2.3.4. Mechanism of Quenching

Based on Equation (3), a possible mechanism has been put forward. The N-nitroso compounds are formed by nitrite and N-CQDs surface amide group under acidic conditions [41], which results in fluorescence static quenching. To prove this hypothesis, N-CQDs were synthesized in aqueous solution by adding different concentrations of NO_2_^−^ for UV-vis detection.

(3) CDs−CONHR+HONO→CDs−CON(NO)R

**Figure 7:**
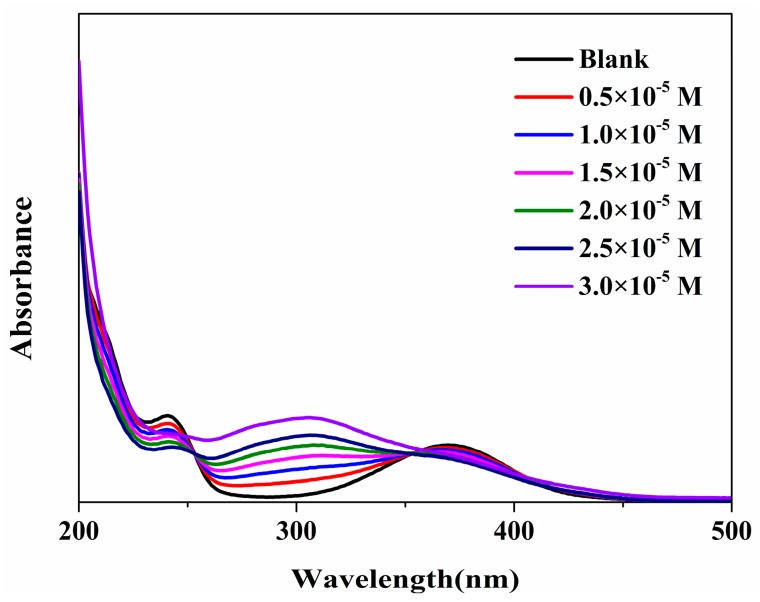
UV-vis. absorption spectra of nitrite at different concentrations added to the N-CQD.

As shown in Figure 7, at a 300 nm wavelength, the absorption increased continuously with the increase of NO_2_^−^ concentration, because the nitroso group was a color enhancing group, which was consistent with the formation of nitroxyl compounds. One method to distinguish static and dynamic quenching is by carefully examining of the absorption spectra of the fluorophore. Collisional quenching only affects the excited states of the fluorophores, thus no changes in the absorption spectra are expected. In contrast, ground-state complex formation will frequently result in perturbation of the absorption spectrum of the fluorophore [42]. This validates the previous conclusions.

### 2.4. Analytical Applications

The detection technique mentioned above was applied to the determination of nitrite in tap water. Prior to the fluorescence assay, freshly collected tap water samples were filtered using membrane separation with a 0.22 μm pore size. The results of the analysis based on standard addition method are shown in Table 2. The recovery of NO_2_^−^ for tap water sample was 98.7–104%, demonstrating that the present measuring method for NO_2_^−^ was credible and applicable to practical applications.

## 3. Materials and Methods

### 3.1. Chemicals/Reagents

Citric acid, sodium nitrite, hydrochloric acid, sodium hydroxide and ethylenediamine were bought from Aladdin Chemical Reagent Co. Ltd. (Shanghai, China). Metal salts (Na_2_CO_3_, Na_2_SO_3_, PbSO_4_, CuCl_2_·2H_2_O, KCl, NaCl, BaCl_2_, HgCl_2_, NaNO_3_, Co(NO_3_)_2_·6H_2_O, Ni(NO_3_)_2_·6H_2_O, Cr(NO_3_)_3_·9H_2_O, FeCl_3_, ZnCl_2_, Na_3_PO_4_, Na_2_HPO_4_, NaH_2_PO_4_, NH_4_F, KBr and KI) were purchased from YongDa Chemical Reagent Co. Ltd. (Tianjin, China). Ultrapure water prepared from a Milli-Q water purification system (Millipore, Billerica, MA, USA) was used throughout the experiments.

### 3.2. Apparatus

A FEI TF-20 instrument operating at 200 kV (FEI, Hillsboro, TX, USA) was used to obtain high resolution transmission microscopy (HRTEM) information. FTIR spectra were collected from 20 scans with a resolution of 4 cm^−1^ by a Magna-IR560 unit (Nicolet Co., Madison, WI, USA). UV-vis. spectroscopy was performed on a UV-2550 spectrophotometer (Shimadzu, Kyoto, Japan) though a quartz cell with a 1 cm optical path. An LS-55 fluorescence spectrometer (PerkinElmer, Waltham, MA, USA) recorded the fluorescence. X-ray photoelectron spectroscopy (XPS) data for the N-CQDs powder deposited on copper substrates were measured by an AXIS Ultra DLD spectrometer (Kratos, Manchester, UK) with a monochromatized Al Kα X-ray source (1486.6 eV) for determining the composition and chemical bonding configurations.

### 3.3. Preparation of N-CQDs

N-CQDs were prepared by hydrothermal treatment of citric acid and EDA. Citric acid (3 g) and EDA (3 mL) were mixed in a tetrafluoroethylene-lined autoclave (50 mL), and water was added until a final volume of 30 mL was reached. The resulting solution was then kept at 180 °C for 5 h. After cooling at room temperature, the mixture was dialyzed using 300 Da cut-off bag with ultrapure water for one day to remove by-products.

### 3.4. NO_2_^−^ Determination

NO_2_^−^ detection is performed using N-CQDs (50 μL, 1.5 mg mL^−1^) with 8 mL HCl-KCl buffer solution and different volumes of NO_2_^−^ stock solution (0.001 M) were added into a 10 mL volumetric flask, and finally diluted with HCl-KCl buffer solution to 10 mL. After thorough mixing, the fluorescence spectra were recorded (equilibrated time 15 min). The NO_2_^−^ selectivity is determined using 100 µL of a single metal ion stock solution (0.001 M) instead of NO_2_^−^ in a similar way.

All the fluorescence detections were under the same conditions: the slit widths of the excitation and emission were both 10 nm, and the fluorescence spectra were recorded at an excitation wavelength of 370 nm with the emission recorded over the wavelength range of 370–600 nm. The fluorescence intensity of the maximum emission peak was used for quantitative and qualitative analysis.

## 4. Conclusions

In this research, the detection of nitrite with N-CQDs has been studied and the optimum concentration of the nitrogen source has been determined. N-CQDs synthesized with EDA (3 mL) as the nitrogen source and citric acid (3 g) as a carbon source were used to detect NO_2_^−^. N-CQDs are introduced as a novel fluorescence probe for NO_2_^−^ detection with a linear range of 0.2–20 μM NO_2_^−^. A possible mechanism has been put forward in that the formation of N-nitroso compounds by nitrite and N-CQDs’ surface amide group under acidic conditions may result in fluorescence static quenching. The method has been applied to the determination of nitrite in tap water with good recovery as well as high reproducibility. This suggests that the N-CQDs can be considered as a suitable fluorescence probe for the determination of NO_2_^−^ without any significant interferences.

## Figures and Tables

**Table 1:** Comparison of LOD and linear range for NO_2_^−^ different CQDs fluorescent probes.

Fluorescent Probe	LOD (nM)	Linear Range (μM)	Ref.
CQDs-NaNO_2_-H_2_O_2_	53	0.1–10	[16]
CQDs-NaNO_2_-H_2_O_2_-Na_2_CO_3_	5	0.1–10	[40]
N-CQDs	25	0.1–75	[31]
N-CQDs	40	0.2–20	[This work]

**Table 2:** Determination of NO_2_^−^ in tap water samples.

Added/μM	Found/μM	Recovery/%
1.00	1.03	103
2.00	2.08	104
3.00	2.96	98.7

## References

[1] Olajos E.J., Coulston F. (1978). Comparative toxicology of n-nitroso compounds and their carcinogenic potential to man. Ecotoxicol. Environ. Saf..

[2] Fan A.M., Nriagu J.O. (2011). Nitrate and nitrite in drinking water: A toxicological review. Encyclopedia of Environmental Health.

[3] Griess P. (2010). Bemerkungen zu der abhandlung der hh. Weselsky und benedikt & bdquo;ueber einige azoverbindungen & rdquo. Eur. J. Inorg. Chem..

[4] Cox R.D., Frank C.W. (1982). Determination of nitrate and nitrite in blood and urine by chemiluminescence. J. Anal. Toxicol..

[5] Garside C. (1982). A chemiluminescent technique for the determination of nanomolar concentrations of nitrate and nitrite in seawater. Mar. Chem..

[6] He D., Zhang Z., Huang Y., Hu Y. (2007). Chemiluminescence microflow injection analysis system on a chip for the determination of nitrite in food. Food Chem..

[7] Badea M., Amine A., Palleschi G., Moscone D., Volpe G., Curulli A. (2001). New electrochemical sensors for detection of nitrites and nitrates. J. Electroanal. Chem..

[8] Zhu N., Xu Q., Li S., Gao H. (2009). Electrochemical determination of nitrite based on poly(amidoamine) dendrimer-modified carbon nanotubes for nitrite oxidation. Electrochem. Commun..

[9] Liu Q.H., Yan X.L., Guo J.C., Wang D.H., Lei L., Yan F.Y., Chen L.G. (2009). Spectrofluorimetric determination of trace nitrite with a novel fluorescent probe. Spectrochim. Acta.

[10] Nakamura M. (2006). Resorcinol as fluorimetric reagent for the determination of nitrate. Anal. Lett..

[11] Rider B.F., Mellon M.G. (1946). Colorimetric determination of nitrites. Ind. Eng. Chem. Anal. Ed..

[12] Armijo F., Goya M.C., Reina M., Canales M.J., Arévalo M.C., Aguirre M.J. (2007). Electrocatalytic oxidation of nitrite to nitrate mediated by Fe(iii) poly-3-aminophenyl porphyrin grown on five different electrode surfaces. J. Mol. Catal. A.

[13] Milhano C., Pletcher D. (2008). The electrodeposition and electrocatalytic properties of copper–palladium alloys. J. Electroanal. Chem..

[14] Silva S.M.D., Mazo L.H. (2015). Differential pulse voltammetric determination of nitrite with gold ultramicroelectrode. Electroanalysis.

[15] Cox R.D. (1980). Determination of nitrate and nitrite at the parts per billion level by chemiluminescence. Anal. Chem..

[16] Lin Z., Xue W., Chen H., Lin J.M. (2011). Peroxynitrous-acid-induced chemiluminescence of fluorescent carbon dots for nitrite sensing. Anal. Chem..

[17] Wang Q.H., Yu L.J., Liu Y., Lin L., Lu R.G., Zhu J.P., He L., Lu Z.L. (2017). Methods for the detection and determination of nitrite and nitrate: A review. Talanta.

[18] Axelrod H.D., Engel N.A. (1975). Fluorometric determination of subnanogram levels of nitrite using 5-aminofluorescein. Anal. Chem..

[19] Hu Y.H., Geng X., Zhang L., Huang Z.M., Ge J., Li Z.H. (2017). Nitrogen-doped carbon dots mediated fluorescent on-off assay for rapid and highly sensitive pyrophosphate and alkaline phosphatase detection. Sci. Rep..

[20] Xu X., Ray R., Gu Y., Ploehn H.J., Gearheart L., Raker K., Scrivens W.A. (2004). Electrophoretic analysis and purification of fluorescent single-walled carbon nanotube fragments. J. Am. Chem. Soc..

[21] Fang Y., Guo S., Li D., Zhu C., Ren W., Dong S., Wang E. (2012). Easy synthesis and imaging applications of cross-linked green fluorescent hollow carbon nanoparticles. ACS Nano.

[22] Li H., Kang Z., Liu Y., Lee S.T. (2012). Carbon nanodots: Synthesis, properties and applications. J. Mater. Chem..

[23] Kong B., Zhu A., Ding C., Zhao X., Li B., Tian Y. (2012). Carbon dot-based inorganic-organic nanosystem for two-photon imaging and biosensing of pH variation in living cells and tissues. Adv. Mater..

[24] Qiang Q., Zhu A., Shao X., Shi G., Yang T. (2012). Development of a carbon quantum dots-based fluorescent Cu^2+^ probe suitable for living cell imaging. Chem. Commun..

[25] Yang S.T., Cao L., Luo P.G., Lu F., Wang X., Wang H., Meziani M.J., Liu Y., Qi G., Sun Y.P. (2009). Carbon dots for optical imaging in vivo. J. Am. Chem. Soc..

[26] Zhao H.X., Liu L.Q., Liu Z.D., Wang Y., Zhao X.J., Huang C.Z. (2011). Highly selective detection of phosphate in very complicated matrixes with an off-on fluorescent probe of europium-adjusted carbon dots. Chem. Commun..

[27] Li H., Zhang Y., Wang L., Tian J., Sun X. (2010). Nucleic acid detection using carbon nanoparticles as a fluorescent sensing platform. Chem. Commun..

[28] Guo Y., Zhang L., Zhang S., Yang Y., Chen X., Zhang M. (2015). Fluorescent carbon nanoparticles for the fluorescent detection of metal ions. Biosens. Bioelectron..

[29] Wang X., Cao L., Lu F., Meziani M.J., Li H., Qi G., Zhou B., Harruff B.A., Kermarrec F., Sun Y.P. (2009). Photoinduced electron transfers with carbon dots. Chem. Commun..

[30] Zhou L., Lin Y., Huang Z., Ren J., Qu X. (2012). Carbon nanodots as fluorescence probes for rapid, sensitive, and label-free detection of Hg^2+^ and biothiols in complex matrices. Chem. Commun..

[31] Doroodmand M.M., Askari M. (2017). Synthesis of a novel nitrogen-doped carbon dot by microwave-assisted carbonization method and its applications as selective probes for optical ph (acidity) sensing in aqueous/nonaqueous media, determination of nitrate/nitrite, and optical recognition of nox gas. Anal. Chim. Acta.

[32] Zhai X., Zhang P., Liu C., Bai T., Li W., Dai L., Liu W. (2012). Highly luminescent carbon nanodots by microwave-assisted pyrolysis. Chem. Commun..

[33] Hu Y., Yang J., Tian J., Yu J.S. (2015). How do nitrogen-doped carbon dots generate from molecular precursors? An investigation of formation mechanism and a solution-based large-scale synthesis. J. Mater. Chem. B.

[34] Zhai Y., Zhu Z., Zhu C., Ren J., Wang E., Dong S. (2014). Multifunctional water-soluble luminescent carbon dots for imaging and Hg^2+^ sensing. J. Mater. Chem. B.

[35] Cai Q.Y., Li J., Ge J., Zhang L., Hu Y.L., Li Z.H., Qu L.B. (2015). A rapid fluorescence “switch-on” assay for glutathione detection by using carbon dots-MnO_2_ nanocomposites. Biosens. Bioelectron..

[36] Qu S., Wang X., Lu Q., Liu X., Wang L. (2012). A biocompatible fluorescent ink based on water-soluble luminescent carbon nanodots. Angew. Chem..

[37] Dong Y.Q., Pang H.C., Yang H.B., Guo C.X., Shao J.W., Chi Y.W., Li C.M., Yu T. (2013). Carbon-based dots co-doped with nitrogen and sulfur for high quantum yield and excitation-independent emission. Angew. Chem. Int. Ed. Engl..

[38] Suzuki H., Iijima K., Moriya A., Mcelroy K., Scobie G., Fyfe V., Mccoll K.E. (2003). Conditions for acid catalysed luminal nitrosation are maximal at the gastric cardia. Gut.

[39] Zhu S., Meng Q., Wang L., Zhang J., Song Y., Jin H., Zhang K., Sun H., Wang H., Yang B. (2013). Highly photoluminescent carbon dots for multicolor patterning, sensors, and bioimaging. Angew. Chem..

[40] Lin Z., Dou X., Li H., Ma Y., Lin J.M. (2015). Nitrite sensing based on the carbon dots-enhanced chemiluminescence from peroxynitrous acid and carbonate. Talanta.

[41] Smith M.B., March J. (2001). March’s Advanced Organic Chemistry.

[42] Eftink M.R., Lakowicz J.R. (2002). Fluorescence Quenching: Theory and Applications. Topics in Fluorescence Spectroscopy.

